# Supplementary figures and images for: Evolutionary Dynamics of Male Reproductive Genes in the Drosophila virilis Subgroup
Source: G3 (Bethesda). 2017 Jul 26;7(9):3145–55. doi: 10.1534/g3.117.1136 (PMC5592939; doi:10.1534/g3.117.1136)

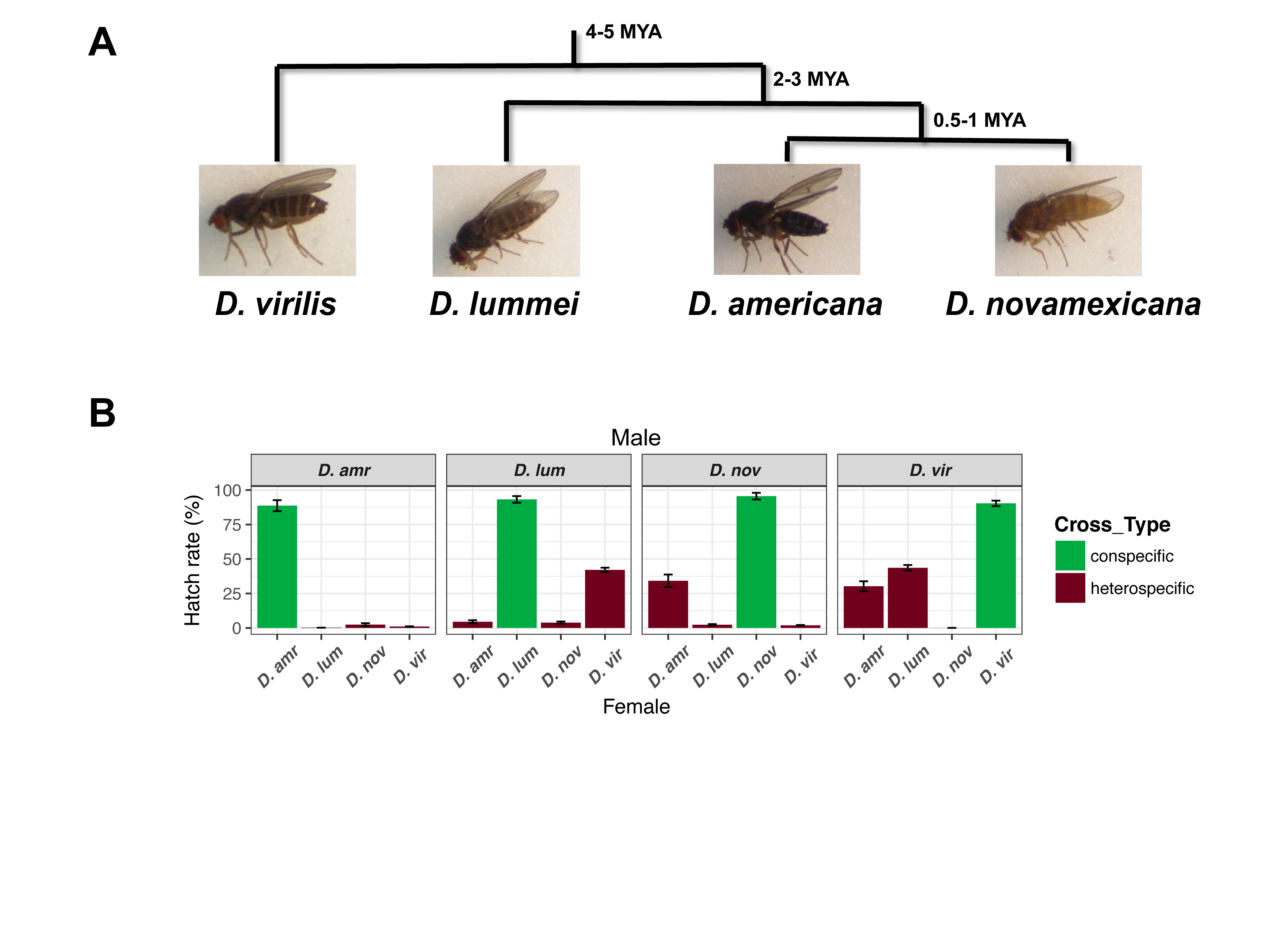

Supplement: Supplementary file 1 [file 3145FigureS1.tif]

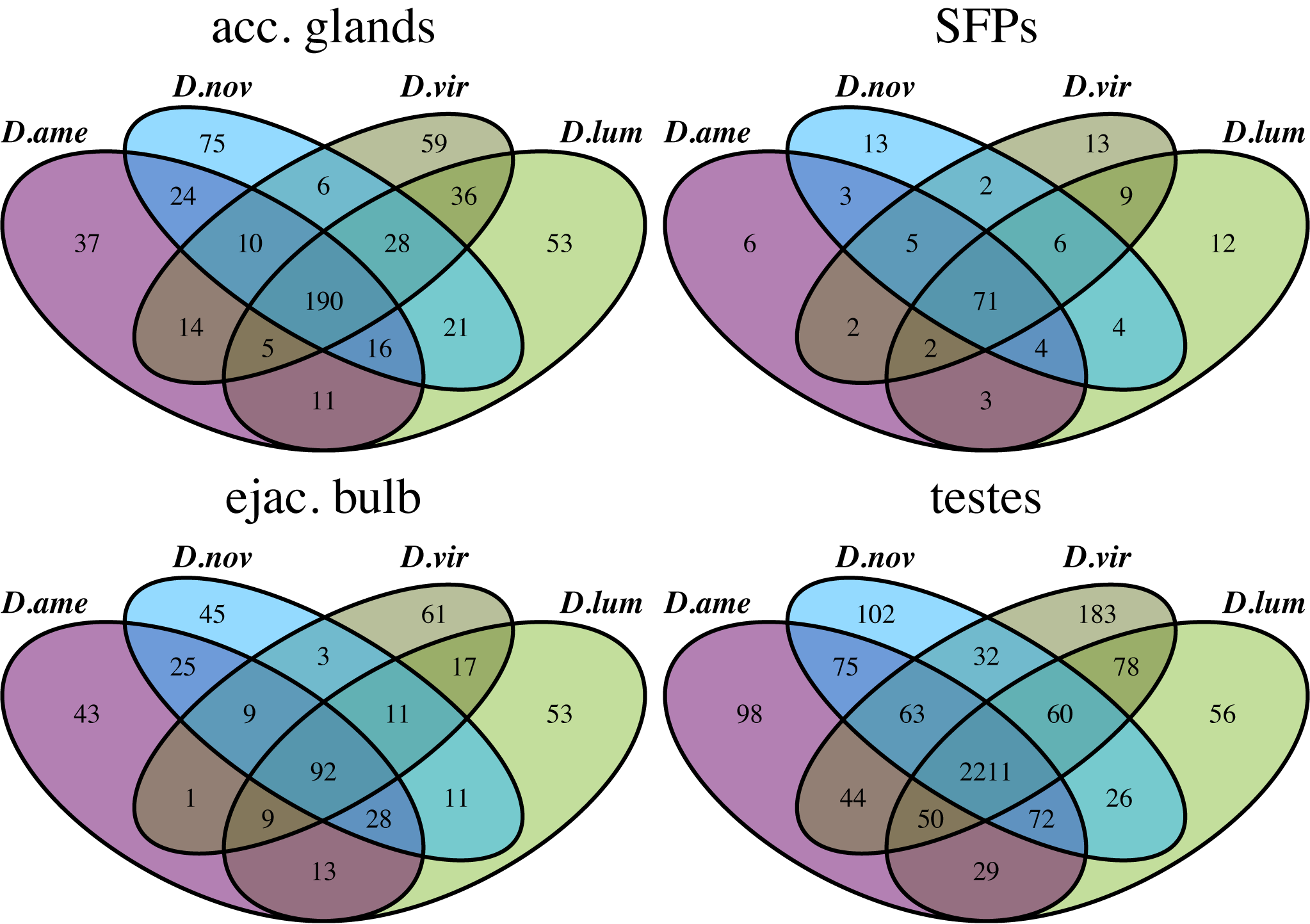

Supplement: Supplementary file 2 [file 3145FigureS2.tif]

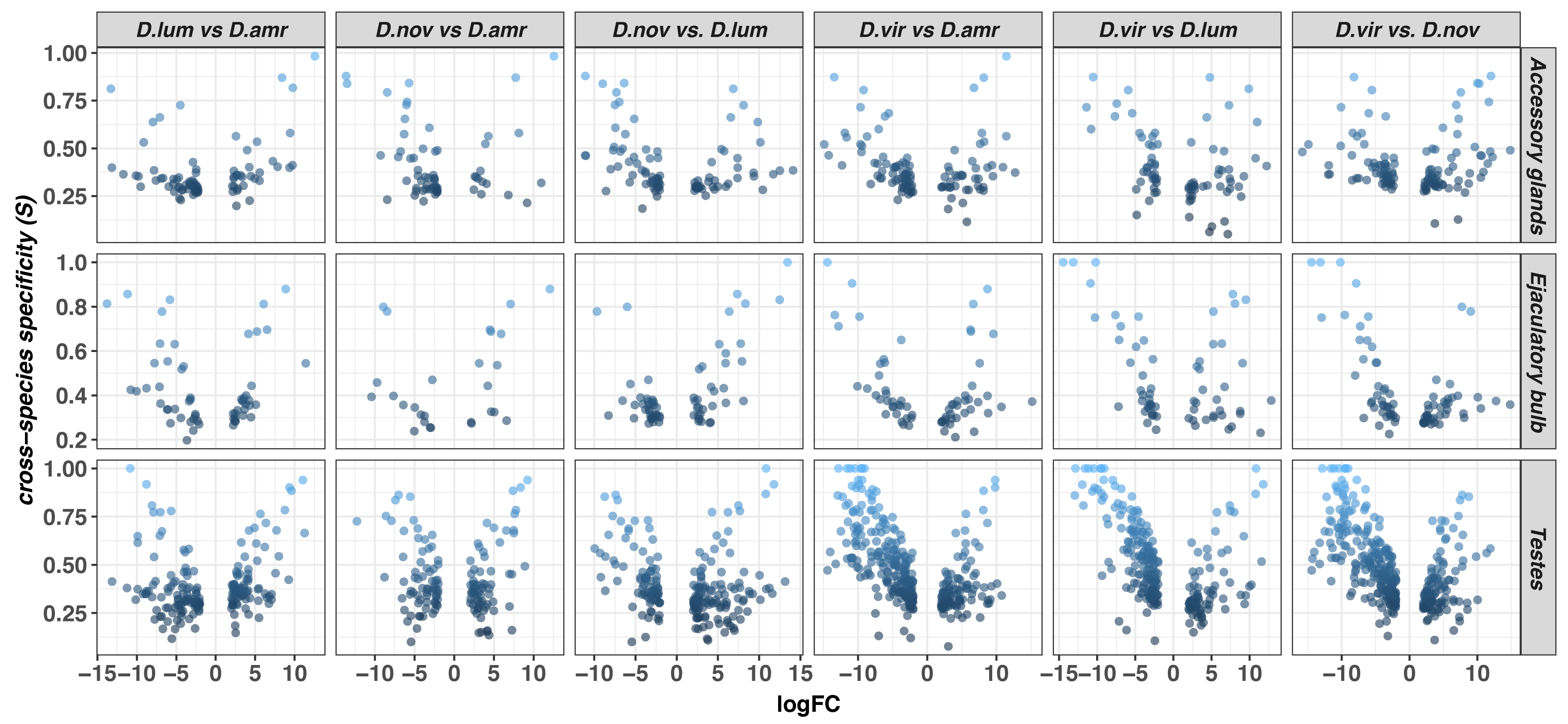

Supplement: Supplementary file 3 [file 3145FigureS3.tif]

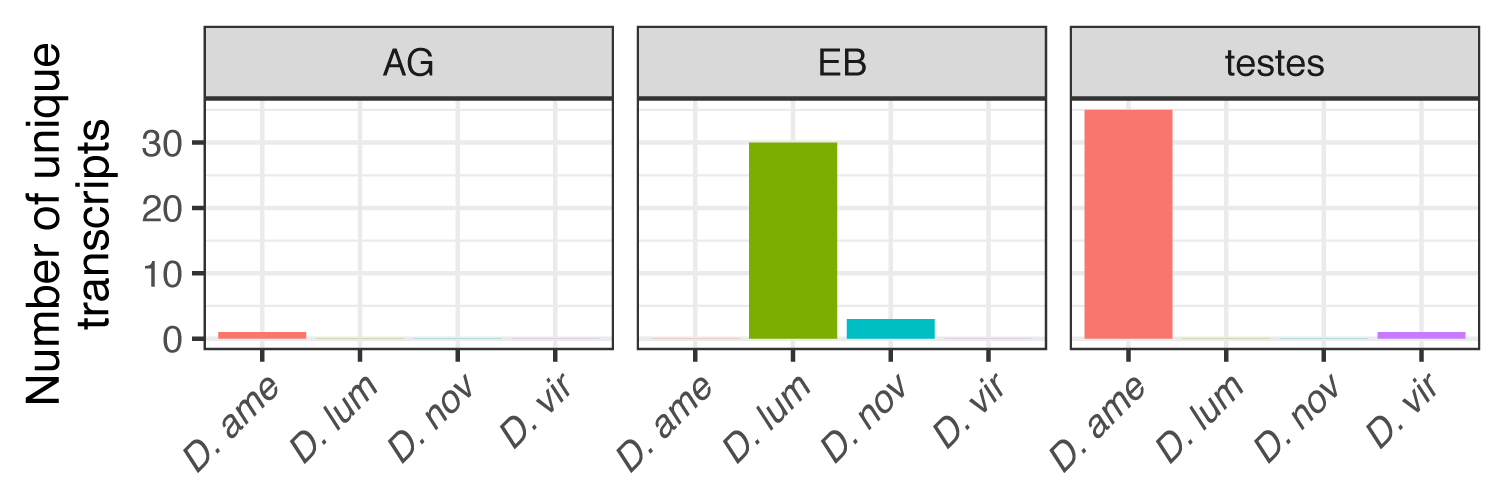

Supplement: Supplementary file 4 [file 3145FigureS4.tif]

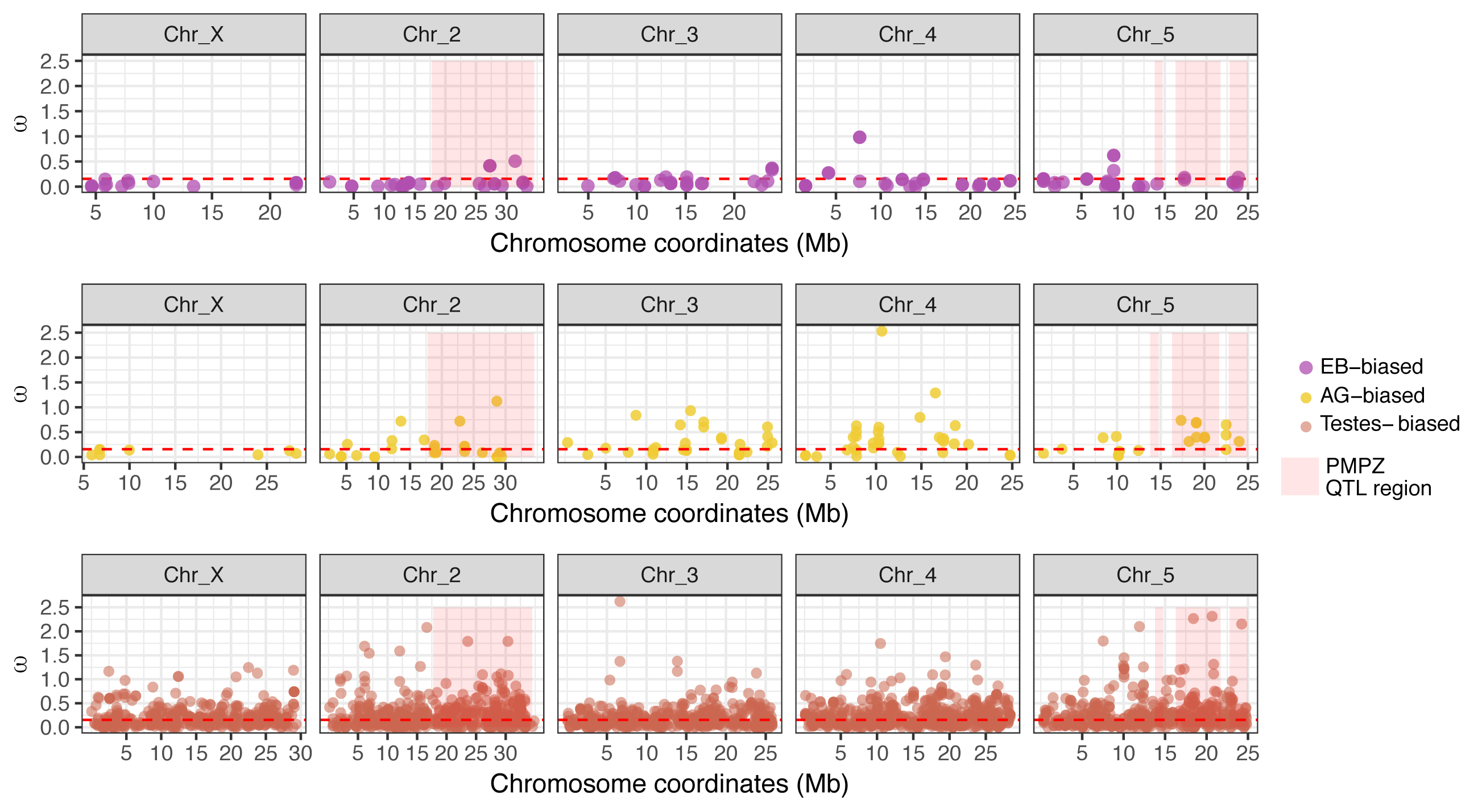

Supplement: Supplementary file 5 [file 3145FigureS5.tif]

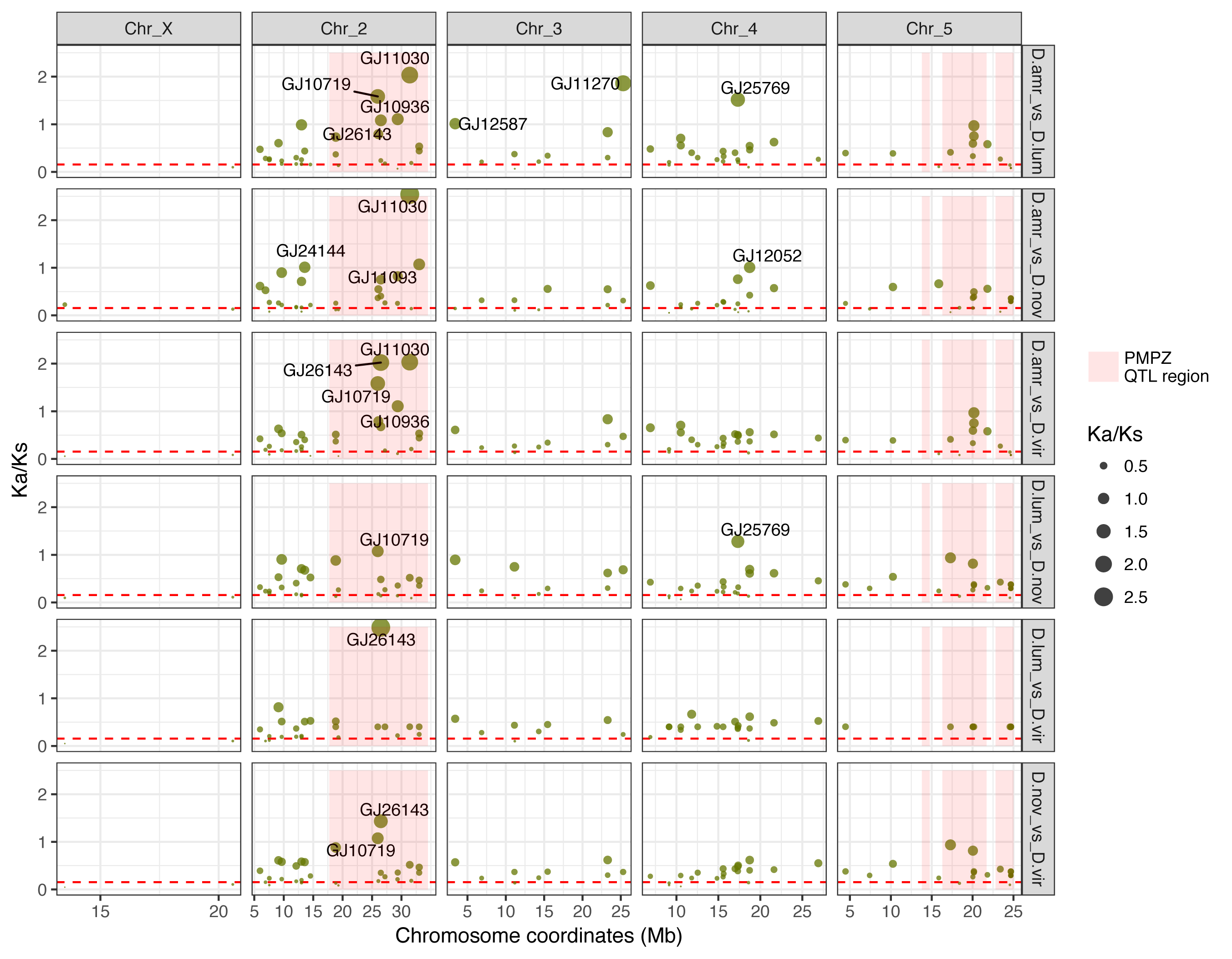

Supplement: Supplementary file 6 [file 3145FigureS6.tif]

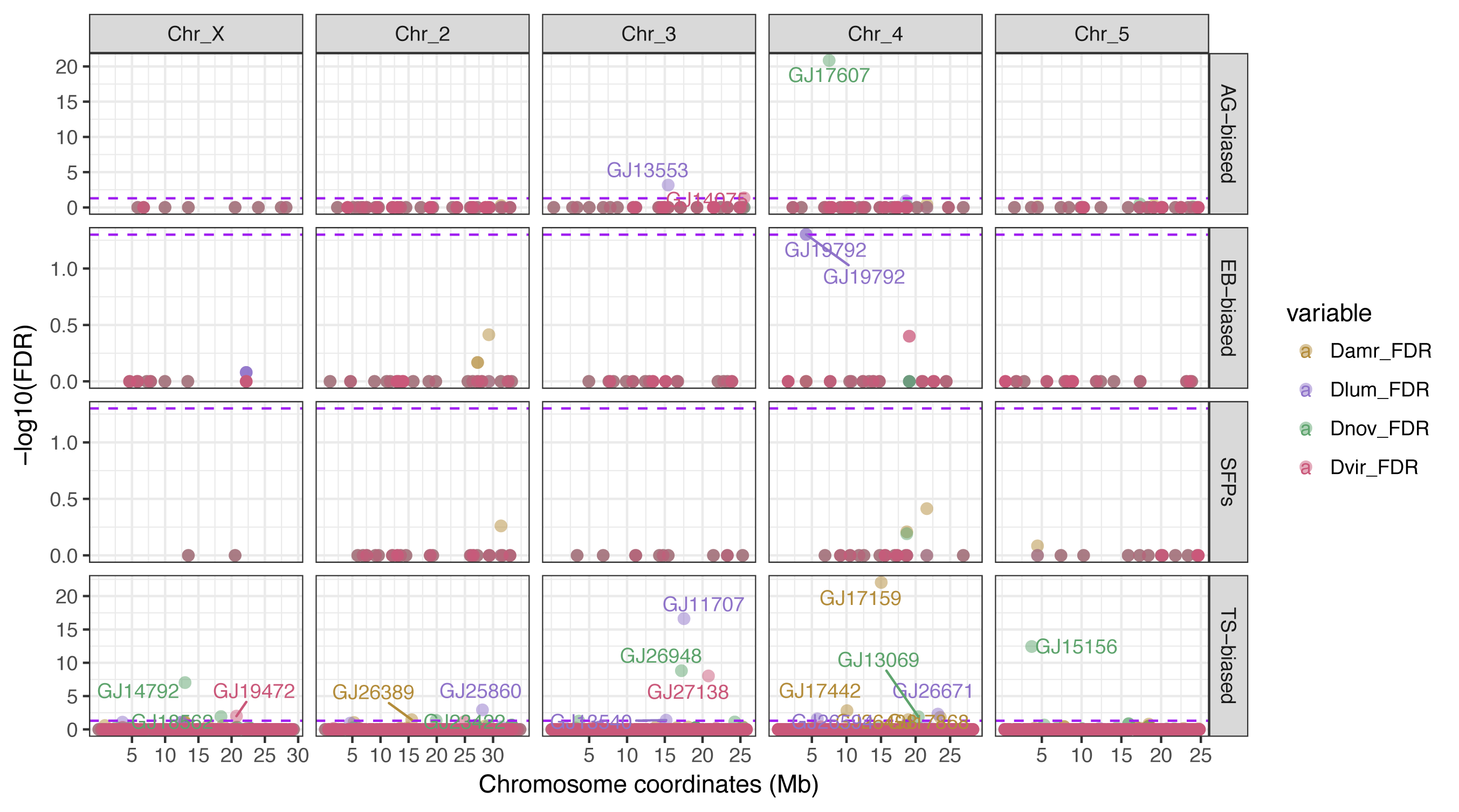

Supplement: Supplementary file 7 [file 3145FigureS7.tif]
